# Intrinsic and Extrinsic Connections of Tet3 Dioxygenase with CXXC Zinc Finger Modules

**DOI:** 10.1371/journal.pone.0062755

**Published:** 2013-05-14

**Authors:** Nan Liu, Mengxi Wang, Wen Deng, Christine S. Schmidt, Weihua Qin, Heinrich Leonhardt, Fabio Spada

**Affiliations:** Department of Biology II and Center for Integrated Protein Science Munich (CIPSM), Ludwig Maximilians University Munich, Planegg-Martinsried, Germany; Université Paris-Diderot, France

## Abstract

Tet proteins are emerging as major epigenetic modulators of cell fate and plasticity. However, little is known about how Tet proteins are targeted to selected genomic loci in distinct biological contexts. Previously, a CXXC-type zinc finger domain in Tet1 was shown to bind CpG-rich DNA sequences. Interestingly, in human and mouse the *Tet2* and *Tet3* genes are adjacent to *Cxxc4* and *Cxxc10-1*, respectively. The CXXC domains encoded by these loci, together with those in *Tet1* and *Cxxc5*, identify a distinct homology group within the CXXC domain family. Here we provide evidence for alternative mouse Tet3 transcripts including the Cxxc10-1 sequence (Tet3^CXXC^) and for an interaction between Tet3 and Cxxc4. *In vitro* Cxxc4 and the isolated CXXC domains of Tet1 and Tet3^CXXC^ bind DNA substrates with similar preference towards the modification state of cytosine at a single CpG site. *In vivo* Tet1 and Tet3 isoforms with and without CXXC domain hydroxylate genomic 5-methylcytosine with similar activity. Relative transcript levels suggest that distinct ratios of Tet3^CXXC^ isoforms and Tet3-Cxxc4 complex may be present in adult tissues. Our data suggest that variable association with CXXC modules may contribute to context specific functions of Tet proteins.

## Introduction

In higher eukaryotes methylation of genomic cytosine to 5-methylcytosine (mC) prominently contributes to epigenetic indexing of transcriptional activity. mC has long been regarded as a stable mark mediating permanent repression, but recent compelling evidence supports a highly dynamic modulation of transcriptional activity by both gain and loss of mC and several pathways for erasure of cytosine methylation have been proposed [1]–[3].

Recently, it has been shown that mC can be progressively oxidized to 5-hydroxymethylcytosine (hmC), 5-formylcytosine (fC) and 5-carboxycytosine (caC) by a three member family of Tet α-chetoglutarate and Fe(II)-dependant dioxygenases [4]–[7]. The discovery of mC derivatives generated by enzymatic oxidation has kindled the idea that they represent intermediates in mC demethylation pathways. Although there is now support for hmC, fC and caC as demethylation intermediates, the relative abundance of hmC in tissues and the stability of its genomic patterns point to a role of this modification as an epigenetic mark with functional relevance distinct from mC [8]–[13]. Direct mutation of Tet2 or inhibition of its catalytic activity by 2-hydroxyglutarate generated through neomorphic IDH1/2 mutations lead to perturbed cytosine methylation patterns in hematopoietic progenitors and are associated with myeloid and lymphoid neoplasia [14]–[17]. Interestingly, Tet1 has been shown to mediate both transcriptional activation and repression and at least part of its repressive function has been proposed to be independent of its catalytic activity [18]–[20]. A role of Tet2 as transcriptional activator has been recently proposed [21], but it is not known whether Tet2 and Tet3 share the dual functional properties of Tet1. Maternally inherited Tet3 has been shown to oxidize paternal genomic mC in the zygote shortly after fertilization and is required for demethylation and subsequent efficient acitivation of the paternal *Oct4* and *Nanog* alleles [22].

Very few interactions involving Tet proteins have so far been reported [18], [20], [23] and even fewer known domains are identified in these proteins despite their relatively large size. As a consequence, little is known about how Tet proteins are targeted to specific genomic loci in distinct cell types and developmental stages. The only relatively well characterized modules in Tet proteins are the double-stranded β-helix fold typical of Fe(II)-dependent oxygenase domains and an N-terminal CXXC-type zinc finger in Tet1, thereby the latter has also been referred to as Cxxc6. The CXXC domains in these proteins, as well as that of Tet1, were shown to bind DNA sequences rich in CpG sites. Similar domains are also present in two factors, Cxxc4 and Cxxc5, shown to antagonize the canonical Wnt pathway and an additional CXXC domain is encoded in Cxxc10-1, a predicted ORF adjacent to the *Tet3* gene [24]–[27]. We have previously shown that the CXXC domains of Tet1, Cxxc4, Cxxc5 and Cxxc10-1 form a distinct homology group among CXXC domains [24]. Although human and mouse Tet3 have also been reported to harbour a CXXC domain in recent reviews [28], [29], experimental evidence for these claims was not available. CXXC domains are present in several other proteins with functions related to DNA and histone modification. Here we provide evidence for *cis* and *trans* association of mouse Tet3 isoforms with Cxxc10-1 and Cxxc4, respectively, and characterize the DNA binding properties of their CXXC domains with respect to the modification state of cytosine at CpG sites. Our data suggest that association with distinct CXXC domains may modulate Tet3 function.

## Results

### Identification and expression pattern of mouse Tet3 transcripts encoding a CXXC domain

**Figure 1:**
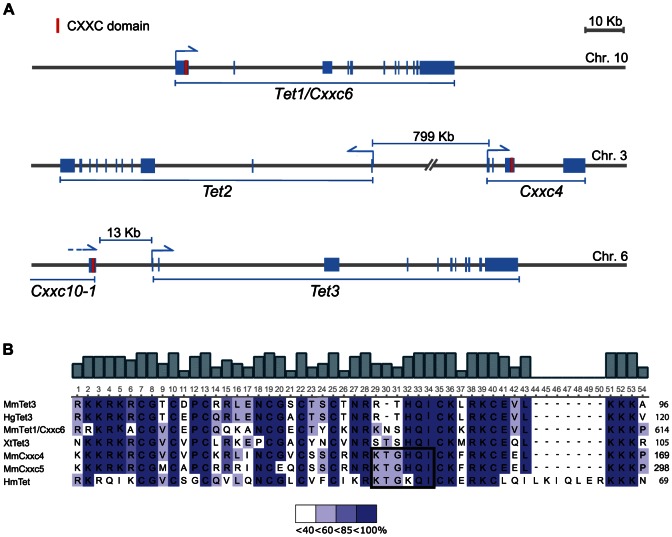
Genomic arrangement of mouse *Tet* genes and adjacent *Cxxc* loci (A) and homology of CXXC domains from mouse Cxxc4, Cxxc5 and Tet homologues in various animal species (B). (A) Schematic representation of mouse *Tet1*, *Tet2/Cxxc4* and *Tet3/Cxxc10* loci. Exons are depicted as blue rectangles. Annotated transcriptional start sites and transcription orientation are indicated with half arrows. (B) Alignment of CXXC domains from mouse Cxxc4, Cxxc5 and Tet homologues in various animal species (Mm, *Mus Musculus*; Hg, *Heterocephalus glaber*; Xt, *Xenopus tropicalis*; Hm, *Hydra mangipallata*). The alignment was generated with Unipro UGENE [64]. Numbers on the right side indicate the position of the last amino acid in the corresponding protein. The KTXXXI motif, previously identified as determinant for the interaction of Cxxc4 with Dvl [54], is boxed (see Discussion). The scale at the bottom indicates the upper limit of percent identity represented by each color. GenBank accession numbers: MmCxxc10, JX946278; XtTet3, NP_001090656.1; HgTet3, EHB01729.1; MmTet1, NP_081660.1; MmCxxc4, NP_001004367; MmCxxc5, NP_598448; HmTet, XP_002161163.1.

The N-terminal region of Tet1 contains a CXXC-type zinc finger domain [4]. In contrast, none of the human and mouse annotated genomic or transcript sequences for Tet2 and Tet3 includes a sequence encoding such domain. However, in both the human and mouse genomes the *Tet2* and *Tet3* genes are adjacent to loci encoding CXXC domains, *Cxxc4* and *Cxxc10-1*, respectively (Fig. 1A) [24], [30]. The *Cxxc4* and *Tet2* loci are 700 and 800 kb apart in the human and mouse genomes, respectively. These loci are transcribed in opposite orientations and encode distinct proteins, suggesting that they evolved through splitting of a *Tet1*-like ancestral gene and intergenic inversion. The Cxxc10-1 ORF was identified *in silico* about 13 kb upstream of the annotated transcriptional start site of *Tet3* and has the same orientation as the Tet3 ORF. Previously, we showed that the CXXC domains of Tet1, Cxxc10-1, Cxxc4 and Cxxc5 constitute a homology group distinct from CXXC domains present in several other factors with functions related to DNA or chromatin modification [24]. The proximity and co-orientation of the Cxxc10-1 and Tet3 ORFs in the human and mouse genomes suggest that alternative Tet3 transcripts may include the Cxxc10-1 ORF. This is also suggested by GenBank entries of Tet3 orthologues encompassing an N-terminal CXXC domain from other vertebrate species, including a *Xenopus* Tet3 transcript and a Tet3 protein homolog predicted from the genomic sequence of the naked mole rat (*Heterocephalus glaber*). Alignment of the CXXC domains from these Tet3 homologues with the CXXC domains of mouse Cxxc10-1, Tet1, Cxxc4 and Cxxc5 shows that they all belong to the same homology subgroup that we identified previously (Fig. 1B). In addition, the *Hydra* genome encodes a single Tet homolog and its predicted protein product contains an N-terminal CXXC domain with key features of this subgroup (Fig. 1B). These observations support the idea of a common ancestral *Tet* gene encoding a CXXC domain and that in addition to Tet1, this arrangement is preserved also in vertebrate Tet3.

**Figure 2:**
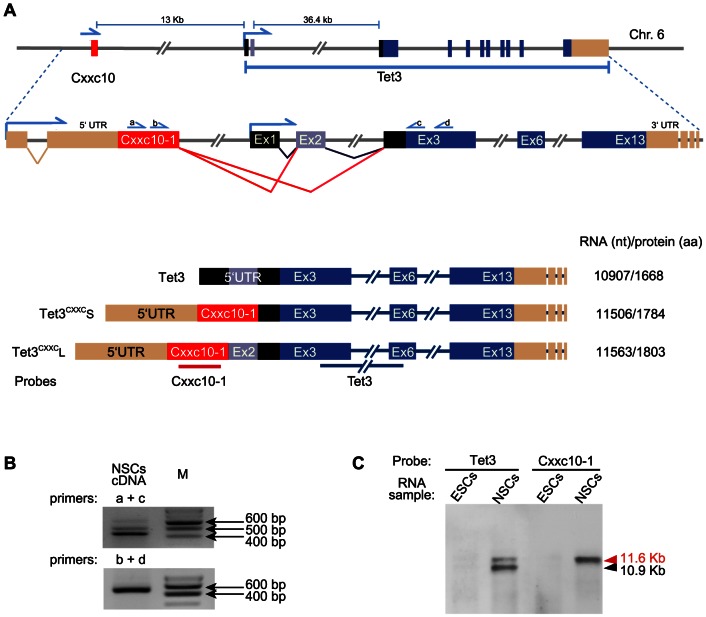
Identification of mouse Tet3 transcript variants encoding a CXXC domain. (A) Drawing illustrating the generation of alternative transcripts from the *Tet3/Cxxc10-1* locus. The positions of primers used in B are reported. The lower part reports a schematic representation of alternative Tet3 transcripts. The positions of the probes used for northern blotting in C are reported. (B) Amplification of fragments from NSCs cDNA identifying Tet3 transcripts that include the Cxxc10-1 ORF. (C) Northern blot detection of alternative Tet3 transcripts in ESCs and NSCs (see Fig. S1 for full and additional blots).

Thus, we set out to verify whether Tet3 transcripts including the Cxxc10-1 ORF are expressed in the mouse. To this aim we performed conventional PCR on total cDNA template from a neural stem cell (NSC) line derived by *in vitro* differentiation of E14 embryonic stem cells (ESCs; Fig. S1). We used primer pairs spanning from the Cxxc10-1 ORF to the Tet3 ORF in exon 3 according to the annotated *Tet3* sequence. Cloning and sequencing of products identified two alternative transcripts where the exon containing the Cxxc10-1 ORF is spliced to the first position of either exon 2 or exon 3 of the annotated *Tet3* gene (Fig. 2A,B). These splicing events set the Cxxc10-1 ORF in frame with the annotated Tet3 coding sequence through its exon 2 and/or exon 3 sequences representing part of the 5′UTR in the annotated Tet3 transcript. Rapid amplification of cDNA 5′ ends (RACE) identified a 5′UTR sequence upstream of the Cxxc10-1 ORF including an additional exon upstream of the one encoding the Cxxc10-1 ORF (Fig. 2A). To verify the expression and size of alternative Tet3 transcripts we first performed northern blotting of RNA from the same NSC line and parental ESCs (Fig. 2D). In NSCs a cDNA probe comprising exons 3–6 of the annotated Tet3 transcript detected two bands with estimated sizes of 10.9 and 11.6 kb, roughly corresponding to the sizes of the annotated Tet3 transcript and those encoding the Cxxc10-1 ORF, respectively, assuming the same splicing events downstream of the annotated exon 3 (Fig. 2A). A probe spanning the Cxxc10-1 ORF detected only the 11.6 kb band. Each of these probes detected the same respective bands in RNA from ESCs, but their intensity was much weaker than for NSCs (not visible in Fig. 2C) despite the same amount of RNA was loaded. We found no evidence for independent expression of the Cxxc10-1 sequence in these samples, as no other distinct band was detected in the blots (Fig. S2). As final evidence for the expression of the Tet3 transcript including the Cxxc10-1 ORF and the annotated exon 2 (hereafter referred to as Tet3^CXXC^L) we amplified its entire coding sequence as a single fragment (5412 bp encoding a polypeptide of 1803 aa) using cDNA from NSCs as template and confirmed its primary structure by sequencing (NCBI accession number JX946278). These results show that the use of an alternative promoter and alternative splicing lead to the expression of Tet3 transcripts containing the Cxxc10-1 ORF (altogether referred to as Tet3^CXXC^) and that these transcripts share the same splicing organization with the previously annotated Tet3 transcript (hereafter referred to as Tet3) downstream of its exons 2 (Tet3^CXXC^L) or 3 (Tet3^CXXC^S; Fig. 2A).

**Figure 3:**
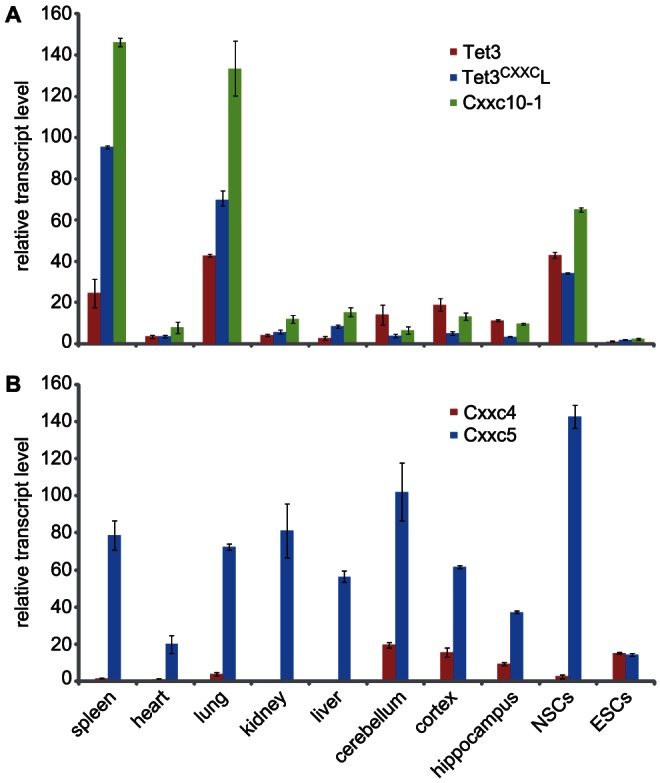
Levels of Tet3, Cxxc4 and Cxx5 transcripts in mouse adult tissues, NSCs and ESCs. Transcript levels were determined by qPCR analysis of total cDNA. (A) Amplfied fragments identify the Tet3 mRNA refseq NM_183138 (Tet3), the alternative Tet3 transcript containing the Cxxc10-1 ORF and exon 2 of NM_183138 (Tet3^CXXC^L) and all transcripts including the Cxxc10-1 ORF. (B) Cxxc4 and Cxx5 transcript levels. Data relative to kidney, liver, cerebellum and cortex samples are from three biological replicates (two 6 week old 129Sv mice and a 30 week old C57BL/6 mouse). Data relative to spleen, heart, lung and hippocampus are from two biological replicates (a 6 week old 129/Sv mouse and a 30 week old C57BL/6 mouse). Data relative to NSCs and ESCs are from three independent cultures each. Shown are mean values and standard errors of the mean (SEM).

To characterize the expression patterns of Tet3 and Tet3^CXXC^ transcripts we performed real time PCR (qPCR) on cDNAs from stem cell lines and various adult mouse tissues (Fig. 3A). We set primer pairs for selective amplification of the Tet3^CXXC^ transcript including exon 2 of the Tet3 transcript, the Cxxc10-1 ORF and exons 1–3 of Tet3. The levels of Tet3 and Tet3^CXXC^ transcripts varied widely across the samples and were very low in ESCs, confirming our northern blot data. Notably, the ratio of Tet3 to Tet3^CXXC^ transcripts was higher in brain regions relative to other tissues.

### Cxxc4 interacts with Tet3 *in vivo* and is expressed in the adult brain

**Figure 4:**
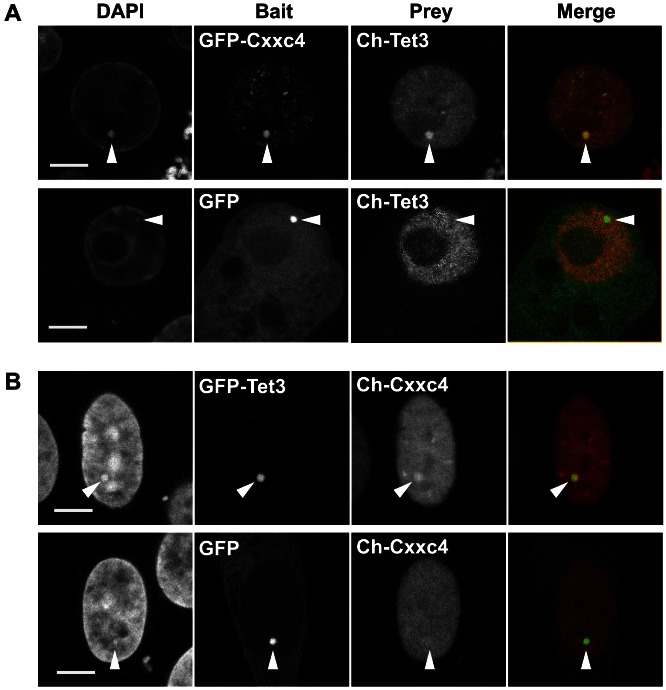
Tet3 and Cxxc4 interact *in vivo*. The interaction was detected by the F3H assay in BHK cells harboring a *lac* operator array (see text and Fig. S2 for explanations). (A) An N-terminal fusion of Tet 3 with Cherry (Ch) was used as prey and GFP-Cxxc4 (upper row) or GFP (as control; lower row) as baits. Localization patterns are representative of 8 (upper row) and 9 (lower row) out of 10 imaged cells. (B) Ch-Cxxc4 was used as prey and GFP-Tet3 (upper row) or GFP (as control; lower row) as baits. Localization patterns are representative of 4 out of 5 (upper row) and 6 out of 7 (lower row) imaged cells. Arrowheads indicate the position of the *lac* operator array as identified by bait signals (GFP channel). Scale bars: 5 µm.

The evolutionary association of Tet proteins with a distinct group of CXXC domains *in cis* raises the question as to whether they associate with this type of CXXC module also *in trans*. Therefore we probed the interaction of each of the three Tet proteins with Cxxc4 and Cxxc5 using a mammalian fluorescent three hybrid assay (F3H). In this assay baits fused to GFP are anchored to a *lac* operator array integrated in the genome of BHK cells and challenged with preys fused to a red fluorescent protein [31]–[33]. The colocalization of prey and bait at the *lac* operator array reflects their interaction (Fig. 4 and Fig. S3). The pair Tet3-Cxxc4 tested positive in both prey-bait combinations, while all other Tet-Cxxc4/5 pairs showed no interaction. However, we could not detect coimmunoprecipitation of Tet3 and Cxxc4 fluorescent fusion constructs overexpressed in HEK293T cells (not shown), which may be due to the lack or limiting endogenous levels of bridging factors in these cells. Cxxc4 and 5 have been shown to antagonize canonical Wnt signaling by binding to cytoplasmic Disheveled [25]–[27]. However, expression of fluorescent fusions revealed a prevalently nuclear localization of Cxxc4 in BHK cells, C2C12 myoblasts and ESCs (Fig. 4 and Fig. S4). In this regard we note that the KKKRK sequence (Fig. 1B) at the N-terminus of the CXXC domain in both Cxxc4 and 5 is a perfect match to the minimal prototypic nuclear localization sequence of the SV40 large T antigen [34], [35], and that Cxxc5 was also found to be predominantly nuclear in various cell types [27], [36].

Next we determined the levels of Cxxc4 and Cxxc5 transcripts in adult mouse tissues and stem cell lines (Fig. 3B). Interestingly, among adult tissues Cxxc4 was expressed mainly in the brain, where Tet3 transcripts that do not encode the CXXC domain were more abundant relative to Tet3^CXXC^ transcripts. In contrast, Cxxc5 mRNA was detected ubiquitously and apart from ESCs its levels were substantially higher than those of Cxxc4. No obvious correlation could be found between the levels of Cxxc5 transcripts and those of any of the Tet transcripts analyzed (Fig. S5).

### The CXXC domains of Tet1, Tet3^CXXC^, CXXC4 and CXXC5 bind CpG containing DNA substrates

Previously, we showed that a construct corresponding to the isolated CXXC domain of mouse Tet1 (aa 561–614) with an N-terminal GFP tag (GFP-CXXC^Tet1^) has very low DNA binding activity *in vitro* [24]. In contrast, Xu *et al.* showed that a larger fragment of mouse Tet1 including the CXXC domain (aa 512–671) binds CpG rich DNA sequences [37]. To resolve this discrepancy we directly compared the DNA binding activity of the isolated CXXC domain of Tet1 with GFP fused either to its N-terminus (the GFP-CXXC^Tet1^ construct we used previously) or to its C-terminus (CXXC^Tet1^-GFP), as well as the same Tet1 fragment used by Xu *et al.* with an N-terminal GFP tag (GFP-Tet1^512–671^; Fig. S6A). These constructs were overexpressed in HEK293T cells, immunopurified and challenged with fluorescent DNA substrates bearing a single CpG site that was either unmodified, symmetrically methylated or symmetrically hydroxymethylated in direct competition [24], [38]–[41]. GFP-Tet1^512–671^ and CXXC^Tet1^-GFP showed similar and substantial binding activity toward substrates containing unmodified and symmetrically methylated CpG sites and were preferred to the substrate with the hydroxymethylated CpG, consistent with previous data [37]. Instead, a much lower DNA binding activity was confirmed for GFP-CXXC^Tet1^ (Fig. S6B). We conclude that the DNA binding properties observed for the Tet1^512–671^ fragment are attributable to the CXXC domain and that direct fusion of GFP at the N-terminus of the isolated CXXC domain interferes with DNA binding.

**Figure 5:**
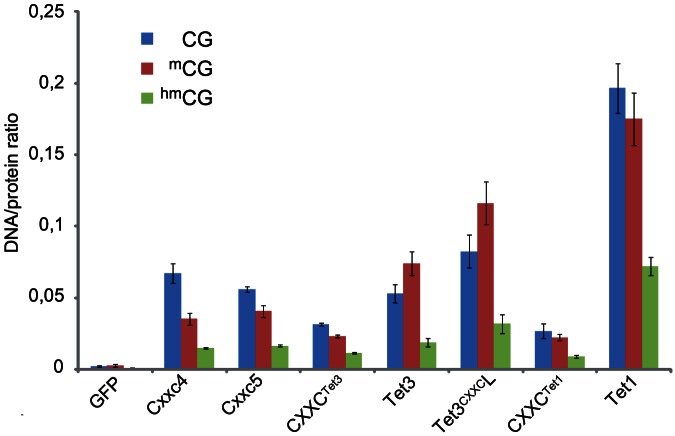
*In vitro* DNA binding properties of Cxxc4 and 5, isolated CXXC domains and full length constructs of Tet1 and Tet3^CXXC^. All proteins were expressed as GFP fusion constructs in HEK293T cells and affinity purified using a GFP-trap. Fluorescently labeled DNA substrates with the same sequence and a single CpG site either unmethylated, symmetrically methylated or symmetrically hydroxymethylated were incubated in direct competition. Shown are mean values of bound substrate/protein ratios and SEM from n independent replicate experiments: Tet1, n = 10; Tet3, CXXC^Tet3^, n = 6; Tet3^CXXC^L, n = 7; CXXC^Tet1^, Cxxc4 and GFP, n = 3; Cxxc5, n = 2.

These results and the high similarity shared by the CXXC domains of Tet1, Tet3^CXXC^ and the Tet3 interactor Cxxc4 prompted us to compare their DNA binding properties. Cxxc4-GFP, Cxxc5-GFP, GFP-Tet1, CXXC^Tet1^-GFP as well as full length Tet1, Tet3 and Tet3^CXXC^L constructs with an N-terminal GFP tag were subjected to similar DNA binding assays as above (Fig. 5 and Fig. S7). CXXC^Tet3^-GFP corresponds to the isolated CXXC domain of the Cxxc10-1 ORF with GFP fused to its C-terminus and is therefore analogous to CXXC^Tet1^-GFP. Although we could not detect interactions between Tet proteins and Cxxc5, we investigated the DNA binding potential of the latter as its CXXC domain is also highly homologous to that of Tet1. CXXC domains belonging to a distinct homology class, including the CXXC domain of Dnmt1 (CXXC^Dnmt1^), were shown to preferentially bind CpG-containing sequences [24], [42]–[46]. Therefore, we first determined the binding preference of our constructs with respect to DNA substrates differing only for the presence or absence of a single central CpG site and compared it to that of the CXXC domain of Dnmt1 (GFP-CXXC^Dnmt1^; Fig. S7). Cxxc4, Cxxc5 and all Tet constructs showed higher DNA binding activity as well as similar and substantial preference for the substrate containing a CpG site as compared to GFP-CXXC^Dnmt1^.We then determined the binding preference with respect to substrates containing a single central CpG site with distinct cytosine modifications as shown above for CXXC^Tet1^ constructs. Cxxc4-GFP, Cxxc5-GFP and CXXC^Tet3^-GFP displayed similar binding properties, with decreasing preference for substrates with the unmodified, symmetrically methylated and symmetrically hydroxymethylated CpG site. In contrast and as shown above, CXXC^Tet1^-GFP did not discriminate between substrates with unmodified and symmetrically methylated CpG. In the case of full length Tet1, Tet3 and Tet3^CXXC^L constructs, incubation with a 4-fold molar excess of DNA substrates is expected to minimize potential competition among multiple DNA binding sites. GFP-Tet1 displayed the same substrate preference as the isolated CXXC domain of Tet1 (CXXC^Tet1^-GFP), albeit with an 8-fold increase in binding activity, indicating that sequences outside the CXXC domain (very likely the catalytic domain) contribute to the affinity for DNA without altering the substrate preference. In contrast, both GFP-Tet3 and GFP-Tet3^CXXC^L showed a relative increase in binding activity toward the substrate with methylated CpG site as compared to CXXC^Tet3^-GFP. Thus, in Tet3^CXXC^L features outside the CXXC domain override the binding preference of the latter.

### Tet3^CXXC^ oxidizes genomic mC *in vivo* and shows slightly lower mobility than the Tet3 isoform lacking the CXXC domain

**Figure 6:**
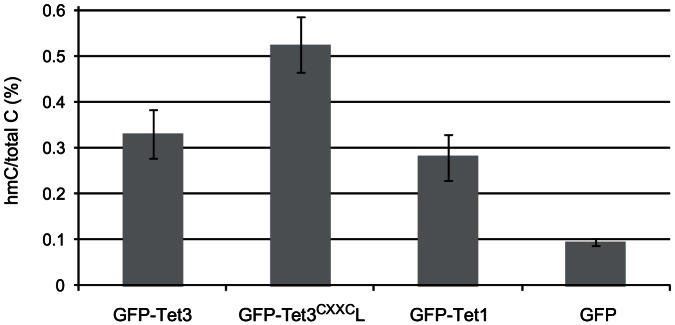
Tet3^CXXC^ oxidizes genomic mC *in vivo*. GFP or GFP-Tet fusions were transiently overexpressed in HEK293T cells and genomic hmC levels were determined using an *in vitro* glucosylation assay with T4 β-glucosyltransferase and UDP-[^3^H]glucose. Shown are mean percentages and SEM of hmC over total C from 2 (GFP-Tet1) or 3 (all others) independent transfections.

We then compared the activity of Tet1 and Tet3 isoforms with or without CXXC domain by determining global levels of genomic hmC in HEK293T cells transiently transfected with GFP-tagged constructs (Fig. 6). A similar increase of hmC levels was observed in cells transfected with GFP-Tet1, GFP-Tet3 and GFP-Tet3^CXXC^L, the latter possibly showing higher conversion of mC to hmC. As further characterization of Tet3 isoforms we compared nuclear localization and mobility of GFP-Tet3 and GFP-Tet3^CXXC^L in C2C12 myoblasts. Both constructs were diffusely distributed throughout the nucleus with exclusion of nucleoli and large clusters of pericentric heterochromatin (chromocenters; Fig. S8A). After photobleaching half of the nucleus the fluorescence of GFP-Tet3^CXXC^L recovered more slowly and reached a plateau at a lower level than that of GFP-Tet3 (Fig. S8B). These differences were small, but reproducible.

Thus, the presence of the CXXC domain in Tet3 does not affect and perhaps promotes conversion of mC to hmC, while it reduces its mobility and slightly increases the immobile fraction, suggesting that the CXXC domain contributes to additional nuclear interactions.

## Discussion

Very limited information is available as to how Tet family dioxygenases target selected genomic loci in distinct developmental and cellular contexts. CXXC-type zinc finger modules have been shown to direct chromatin modifying activities, including Tet1, to CpG rich sequences where they contribute to the establishment of a transcriptionally competent environment [37], [46]–[48]. We now provide evidence that alternative mouse Tet3 isoforms associate with distinct CXXC modules also endowed with DNA binding activity. Alternative presence of an intrinsic CXXC domain or interaction with Cxxc4 may constitute the basis for differential targeting of Tet3 isoforms. In this regard we note that the ratio of Tet3 to Tet^CXXC^ transcripts was higher in brain tissues where Cxxc4 transcripts were more abundant. However, we found that *in vitro* Cxxc4 and the CXXC domain of Tet3^CXXC^ isoforms have similar binding preference with respect to the modification state of cytosine at CpG sites and that DNA binding elements other than the CXXC domain dominate the global DNA substrate preference of Tet3^CXXC^. Further investigation is required to assess how DNA binding by Cxxc4 and the CXXC domain of Tet3^CXXC^ contribute to Tet3 function *in vivo*.

While the current manuscript was under review a report was published showing a role for CXXC domain-containing Tet3 orthologues in early neural and eye development of Xenopus [49]. In the same publication the cloning of human and mouse Tet3 isoforms containing a CXXC domain was reported, the latter being identical to our mouse Tet3^CXXC^L, but no expression or functional data were provided for these mammalian isoforms. Importantly, their isothermal titration calorimetry data on the DNA binding properties of the CXXC domain from *Xenopus* and human TET3 isoforms are fully consistent with the results of our DNA binding assays with the CXXC domain of mouse Tet3^CXXC^.

Association with distinct CXXC domains may also modulate Tet protein function by additional mechanisms. Interestingly, Cxxc4 and Cxxc5 were shown to antagonize Wnt signaling by competing with Axin for binding to Dishevelled (Dvl), thus leading to destabilization of β-catenin [25]–[27]. Although β-catenin stabilization by Dvl occurs in the cytoplasm, nuclear Dvl has been shown to interact with a two megadalton TCF/β-catenin transcriptional complex and to be required for activation of Wnt pathway target genes [34], [50], [51]. Importantly, we found that Cxxc4, like Cxxc5, is predominantly nuclear. Interestingly, other factors interacting with Dvl such as DP1 and NFAT are known to shuttle between cytoplasm and nucleus [52], [53]. DP1 was shown to play dual regulatory roles in Wnt signaling depending on its nucleocytoplasmic localization, while dephosphorylated NFAT was proposed to inhibit canonical Wnt signaling by sequestering Dvl from transcriptional complexes in the nucleus. Therefore, it will be interesting to investigate whether Cxxc4 and Tet3 are involved in nuclear TCF/β-catenin complexes and affect transcription of their target genes. A KTXXXI motif within the CXXC domain of Cxxc4 was previously shown to be minimally required for the interaction with Dvl [54], but is poorly conserved in the CXXC domain of vertebrate Tet3^CXXC^ isoforms (Fig. 1B). Differential expression of Tet3 isoforms and interaction with Cxxc4 may therefore modulate the recruitment of Tet3 to TCF/β-catenin complexes. Thus, our results warrant further investigation on the functional relevance of the association between Tet proteins and CXXC modules.

## Materials and Methods

### Ethics statement

Collection of animal tissues was performed in accordance with the German Animal Protection Law. No experiment was performed on live animals. Mice were painlessly killed under anesthesia with Isofuran before harvesting organs and tissues. According to the German Animal Welfare Act (Part III: “Killing of animals”, Section 4, May 18, 2006) postmortem collection of tissues and organs does only require summary notification to the animal protection institution, but does not require any special permission. Therefore, this study was not registered as an animal experiment and the animal tissues used are registered only in the annual report of animals sacrificed for research and study to the relevant authority.

### Cell culture

E14 [55] and CGR8 [56] ESCs were maintained in gelatin coated flasks with DMEM high glucose containing 16% FBS, 2 mM L-glutamine, 100 U/ml penicillin, 100 µg/ml streptomycin (all from PAA Laboratories GmbH), 1× MEM Non-essential Amino Acid Solution and 0.1 mM β-mercaptoethanol (both from Invitrogen) and supplemented with 3 µM CHIR 99021 and 1 µM PD0325901 (“2i”; both from Axon Medchem). The NSC line ENC1 used throughout this study was derived from E14 ESCs as described [57] and was maintained in Knockout-DMEM/F12 containing 2 mM GlutaMAX-I (both from Invitrogen) 100 U/ml penicillin, 100 µg/ml streptomycin, and supplemented with 1% N2 (custom made according to [58]) and 20 ng/ml each FGF-2 and EGF (PeproTech). ENC1 cells homogeneously expressed NSC markers Nestin, Pax6 and Olig2 (Fig. S1). C2C12 myoblasts [59] HEK293T [60] cells and BHK cells with a stably integrated *lac* operator array [61] were cultured as described [24], [32], [33].

### Expression constructs

Throughout this study enhanced GFP and monomeric Cherry fusion constructs were used and are referred to as GFP and Cherry fusions, respectively, for brevity. GFP-Tet1 and GFP-CXXC^Tet1^ were described previously [24]. For other GFP and Cherry fusions cDNA was generated from either ENC1 NSCs (Tet3, Tet3^CXXC^L, CXXC^Tet3^, Cxxc5) or parental E14 ESCs (Cxxc4) with the RevertAid Premium First Strand cDNA Synthesis kit (Thermo Scientific). Coding sequences were amplified using Phusion High-Fidelity DNA polymerase (New England Biolabs) and primers listed in Table S1. Sequences coding for Tet3, Tet3^CXXC^L and Tet1^512–671^ were inserted into the pCAG-GFP-IB vector [62] or the derived pCAG-Cherry-IB vector to generate N-terminal GFP and Cherry fusions, respectively. Sequences coding for CXXC^Tet1^ CXXC^Tet3^, Cxxc4 and Cxxc5 were inserted into pCAG-Tev-GFP (derived from pCAG-GFP-IB) to generate C-terminal GFP fusions. Cxxc4 and Cxxc5 coding sequences were also inserted into pCAG-Cherry-IB to generate N-terminal Cherry fusions. All constructs were verified by DNA sequencing and their expression by western blotting (Fig. S9).

### Northern blotting, cDNA synthesis and qPCR

Total RNA was extracted using the NucleoSpin Triprep Kit and the poly(A)^+^ fraction was enriched with the Nucleotrap mRNA Mini kit (both from Macherey-Nagel). Northern blotting was performed according to the DIG Application Manual for Filter Hybridization (Roche). Probes were generated and labeled by PCR using DIG-dUTP and primers listed in Table S2. Ten micrograms each of total RNA from ESCs and NSCs were separated on formaldehyde-agarose gels, transferred to Hybond-N+ nylon membranes (GE healthcare) and immobilized by UV crosslinking. Blots were prehybridized with DIG Easy hyb (Roche) at 50°C for 30 min followed by overnight hybridization at 50°C. Probes were applied at a final concentration of 100 ng/ml in DIG Easy hyb. After washing, the blots were incubated with blocking solution (Roche) for 30 min, followed by incubation with alkaline phosphatase conjugated anti-digoxygenin antibody (Roche) for 30 min at room temperature. The membrane was washed twice, equilibrated with detection buffer (0.1 M Tris-HCl, 0.1 M NaCl, pH 9.5) and chemiluminescence with CDP-Star substrate (Roche) was used to detect the bound antibody.

Tissue samples were prepared from 6 week old 129Sv and 30 week old C57BL/6 mice (see legend to Fig. 3 for details). Total RNA (500 ng) was reverse transcribed with High-Capacity cDNA Reverse Transcription Kit (Applied Biosystems) according to the manufacturer's instruction. Primers for conventional PCR indicated in Fig. 2A,B are listed in Table S2. Real-time PCR was performed using Power SYBR Green PCR Master Mix (Applied Biosystems) on a 7500 Fast Real-Time PCR System (Applied Biosystems) with primers listed in Table S3. Glyceraldehyde phosphate dehydrogenase (GAPDH) was used for normalization and the comparative CT method was used to analyze expression data.

### 5′ RACE

5′ RACE was performed as described [63] and primers are listed in Table S2. Briefly, 100 ng of total RNA from ENC1 NSCs were reverse transcribed as described above, but using the gene-specific primer1 (GSP1). To remove excess primer, the reaction was purified with a silica mini-column (Nucleospin Gel and PCR Clean-up; Macherey-Nagel). After tailing with terminal deoxynucleotide transferase and dATP the tailed cDNA was subjected to nested PCR reactions with Phusion High-Fidelity DNA Polymerase (New England Biolabs). In the first reaction the upstream primers were (dT)_17_-adaptor primer and adaptor primer, while the downstream primer was gene-specific primer2 (GSP2). Cycling parameters were as follows: one cycle of 98°C for 30 s, 94°C for 5 min, 50°C for 5 min, and 72°C for 40 min, followed by 30 cycles of 94°C for 40 s, 54°C for 1 min, and 72°C for 3 min, with a final cycle of 94°C for 40 s, 54°C for 1 min, and 72°C for 15 min. In the second reaction the upstream primer was adaptor primer and the downstream primer was gene specific primer 3 (GSP3). Cycling parameters were as follows: 98°C for 30 s, (98°C for 15 s, 55°C for 20 s, and 72°C for 30 s) 30 cycles, 72°C for 10 min. PCR products were purified by gel electrophoresis followed by silica column purification, cloned into pCR-Blunt with Zero Blunt PCR Cloning Kit (Invitrogen) and analyzed by sequencing.

### F3H assay

F3H assay (Fig. S3) was performed as described [33]. Briefly, BHK cells with a stably integrated *lac* operator array [61] were seeded on coverslips, cotransfected with GFP binding protein (GBP)-lacI, GFP-bait and Ch-prey constructs, fixed and imaged 16 h after transfection.

### *In vitro* DNA binding assay

*In vitro* DNA binding assays were performed as described previously [24], [38], [39]. Briefly, two or three double stranded DNA oligonucleotides labeled with different ATTO fluorophores were used as substrates in direct competition. DNA oligonucleotide substrates with identical sequence contained an unmodified, symmetrically methylated or symmetrically hydroxymethylated cytosine at a single, central CpG site (CG, mCG and hmCG substartes), while the noCG substrate contained a TpG site at the same position and had otherwise the same sequence (Tables S4, S5, and S6). GFP fusion constructs were expressed in HEK293T cells by transient transfection and immunopurified from cell lysates using the GFP-trap (ChromoTek). GFP-trap beads were washed three times before incubating with DNA substrates at a final concentration of 160 nM each. After removal of unbound substrates, protein amounts (GFP fluorescence) and bound DNA were measured with an Infinite M1000 plate reader (Tecan).

### Determination of global genomic hmC levels

Global hmC levels in genomic DNA from transiently transfected HEK293T cells were determined by the *in vitro* glucosylation assay as described previously [11], [24] with minor modifications. Briefly, 50 µl reactions containing 150 mM NaCl, 20 mM Tris, pH 8.0, 25 mM CaCl2, 1 mM DTT, 3.5 µM UDP-[^3^H]glucose (20 Ci/mmol; Hartmann Analytic GmbH), 500 ng of sheared genomic DNA and 40 nM recombinant T4 β-glucosyltransferase were incubated for 20 min at room temperature and terminated by heating at 65°C for 10 min. DNA fragments were purified by silica column chromatography (Nucleospin, Macherey-Nagel) and radioactivity was determined by liquid scintillation. Radioactive counts were converted to percentages of hmC over total C using curves from PCR generated standards containing variable hmC/C ratios as previously described [11]. The values for all GFP-Tet constructs were corrected for differences in expression levels using GFP-fluorescence measurements. This correction was not applied to control samples transfected with GFP as the latter is expressed at least at ten times higher levels than GFP-Tet1 constructs, which would lead to artificially enhanced differences between basal hmC levels and those resulting by overexpression of Tet constructs.

## Supporting Information

Figure S1 **Expression of NSCs markers in ENC1 cells.** Epifluorescence images of immunofluorescent stainings with antibodies to the indicated markers. Antibody sources: Nestin, mouse monoclonal antibody Rat-401 (Developmental Studies Hybridoma Bank, University of Iowa); Pax6, rabbit polyclonal antibody (PRB-278P, Covance). Olig2, rabbit polyclonal antibody (AB9610, Millipore). Scale bars: 10 µm.
(EPS)

Figure S2 **Northern blot analysis of Tet3 and Tet3^CXXC^L transcripts in NSCs and ESCs (related to** **Fig. 2** **).** On the right the same blot as in Fig. 2D is shown uncropped. In this blot total RNA was loaded [without poly(A)^+^ enrichment], resulting in stronger crosshybridization with 28S and 18S ribosomal RNAs.
(EPS)

Figure S3 **Schematic representation of the mammalian F3H assay (related to** **Fig. 4** **).**
(EPS)

Figure S4 **Nuclear localization of GFP-Cxxc4 in C2C12 myoblasts and CGR8 ESCs (related to** **Fig. 4** **).** Epifluorescence images of transiently transfected cells. Scale bars: 5 µm.
(EPS)

Figure S5 **Transcript levels of Cxxc4, Cxx5 and Tet1–3 in adult mouse tissues ESCs and NSCs (related to** **Fig. 3** **).** In (A) the same plot as in Fig. 3B is reported for ease of comparison between transcript levels of Cxxc4/5 (A) and Tet1–3 (B). In (B) cumulative levels of all Tet3 transcripts were determined using a primer set spanning common sequences downstream exon 3 of the annotated *Tet3* gene. Shown are mean values and SEM. Sample sources and replicates are as for Fig. 3.
(EPS)

Figure S6 ***In vitro*** **DNA binding properties of GFP-Tet1^512–671^, GFP-CXXC^Tet1^ and CXXC^Tet1^-GFP.** (A) Schematic representation of assayed Tet1 constructs. Start and end positions relative to full length Tet1 protein are reported. (B) DNA binding assay as in Fig. 5. Shown are mean values and SEM from 4 independent experiments.
(EPS)

Figure S7 ***In vitro*** **binding of various full length Cxxc domain-containing proteins and isolated CXXC domains to DNA substrates containing one or no CG site), but otherwise identical sequence (related to** **Fig. 5** **).** All constructs are GFP fusions. Shown are mean values of bound substrate/protein ratios and SEM from n independent replicate experiments: GFP and CXXC^Tet3^-GFP, n = 5; GFP-Tet1, Cxxc4-GFP, Cxxc5-GFP and GFP-CXXC^Dnmt1^, n = 4; GFP-Tet3, GFP-Tet3^CXXC^L and CXXC^Tet1^-GFP, n = 3.
(EPS)

Figure S8 **Localization and mobility of Tet3 and Tet3^CXXC^L isoforms in C2C12 nuclei.** (A) Optical sections of fixed C2C12 cotransfected with GFP-Tet3^CXXC^L and Ch-Tet3 constructs as indicated. Arrowheads indicate the position of large chromocenters from which GFP-Tet3^CXXC^L and Ch-Tet3 signals are excluded. (B) FRAP curves of GFP-Tet3 and GFP-Tet3^CXXC^L in transiently transfected C2C12 myoblasts. Images were taken every 150 ms in the first 60 s, and then at intervals of 1 s for the next 120 s. Shown are mean values and SEM from 12 (GFP-Tet3) and 10 cells (GFP-Tet3^CXXC^L). Live cell imaging and FRAP analysis was performed as described (Schermelleh et al., 2007, Nucl Acids Res 35: 4301) with the following minor modifications. The images were Gauss-filtered (2 pixel radius) and data sets showing lateral movement were corrected by image registration using the StackReg plug-in of ImageJ, starting with a time frame where approximately half recovery was reached.
(EPS)

Figure S9 **Western blot analysis of fluorescent fusion proteins.** (A) GFP-CXXC^Dnmt1^, CXXC^Tet3^-GFP, CXXC^Tet1^-GFP, Cxxc4-GFP, Cxxc5-GFP. (B) GFP-Cxx4 and GFP-Cxxc5. (C) GFP-Tet1, GFP-Tet3 and GFP-Tet3^CXXC^L. (D) Cherry-Tet3. Blots were probed with an anti-GFP antibody (A–C) or with an anti-RFP antibody recognizing an epitope present in both RFP and Cherry (D). In all cases the major reacting band migrated as a peptide with the expected mass of the specific, full length fluorescence fusion and in no case peptides with mass corresponding to the fluorescent protein moiety (GFP or Cherry) were detected.
(EPS)

Table S1 **Primer sequences for cloning of coding sequences in expression constructs.**
(DOCX)

Table S2 **Primer sequences for 5′ RACE, conventional RT-PCR, northern blotting probes.**
(DOCX)

Table S3 **Primer sequences for qPCR.**
(DOCX)

Table S4 **Sequences of oligonucleotides used for preparation of double stranded DNA substrates.**
(DOCX)

Table S5 **CG, mCG and hmCG containing DNA substrates used for** ***in vitro*** **binding assay (related to** **Fig. 5** **).**
(DOCX)

Table S6 **CG and noCG containing DNA substrates used for** ***in vitro*** **binding assay (related to Fig. S7).**
(DOCX)

Combined Supporting Information File S1 (PDF)
