# Supplementary material for: Intrinsic and Extrinsic Connections of Tet3 Dioxygenase with CXXC Zinc Finger Modules
Source: PLoS One. 2013 May 14;8(5):e62755. doi: 10.1371/journal.pone.0062755 (PMC3653909; doi:10.1371/journal.pone.0062755)
Supplement: Table S1 — Primer sequences for cloning of coding sequences in expression constructs. (DOCX) [file pone.0062755.s010.docx]

**Table S1.** Primers for amplification and insertion of coding sequences in expression constructs.

| Construct | Primer |
| --- | --- |
| Tet1^512-671^ | 5’-AAG CGA TCG CTT AGA TCT TAC CCA GGG-3’ |
|  | 5’-TTG CGG CCG CCA AAT CCA ACC TTT GC-3’ |
| CXXC^Tet1^ | 5’-GGC GAT CGC ATG TCT ACG CCG CCA ATG-3’ |
|  | 5’-CGC GGC CGC CTG GCT TCT TTT TGA GCA-3’ |
| Cxxc4 | 5′-ATG CAC CAC CGG AAC GAC TCC CAG CG-3’ |
|  | 5’-TTA AAA GAA CCA TCG GAA CGC TTC AGC-3’ |
| Cxxc5 | 5′-AAG CGA TCG CAT GTC GAG CCT CGG CGG TGG-3′ |
|  | 5′-GCG CGG CCG CTC ACT GAA ACC ACC GGA AGG-3′ |
| CXXC^Tet3^ | 5′-ATG CGA TCG CAT GCT GCG AGG GGG TGG AGA T-3′ |
|  | 5′-ATG CGG CCG CCC GCT TTT TTC TTC AGC ACC TC-3′ |
| Tet3^CXXC^L | 5′-GGG CGA TCG CAT GAG CCA GTT TCA GGT GCC CTT GG-3′ |
|  | 5′-GCG GCC GCC TAG ATC CAG CGG CTG TAG GGG CC-3′ |
