# Supplementary material for: Intrinsic and Extrinsic Connections of Tet3 Dioxygenase with CXXC Zinc Finger Modules
Source: PLoS One. 2013 May 14;8(5):e62755. doi: 10.1371/journal.pone.0062755 (PMC3653909; doi:10.1371/journal.pone.0062755)
Supplement: Table S2 — Primer sequences for 5′ RACE, conventional RT-PCR, northern blotting probes. (DOCX) [file pone.0062755.s011.docx]

**Table S2.** Primer sequences for 5′ RACE, conventional RT‑PCR (primers a-d indicated in Fig. 2A,B) and generation of probes for northern blotting.

| Name | Sequence |
| --- | --- |
| GSP1 | 5′ -AGG TCC ATC AAC TGG GCT*-*3′ |
| (dT)_17_-adaptor | 5′-GAC TCG AGT CGA CAT CGA (T)_17_-3′ |
| adaptor primer | 5′-GAC TCG AGT CGA CAT CG-3′ |
| GSP2 | 5′-AGC ACC TCA CAC TTG CG-3′ |
| GSP3 | 5′-GCA GCT GGT ACA AGA CC-3′. |
| Primer a | 5′- GCG ATC GCA TGA GCC AGT TTC AGG -3′ |
| Primer c | 5′- AAG CGG CCG CCA GTC GGG CTT CTG GTC TAC -3′ |
| Primer b | 5′- ATG GCT GGG AGT GAG AC -3′ |
| Primer d | 5′- ATC GCA GGT GCA GTT GGG TG -3′ |
| CXXC10 probe for | 5′-CAC ACC CAT TGG CTC ACC T-3′ |
| CXXC10 probe rev | 5′-GGG TCT CAC TCC CAG CCA-3′ |
| Tet3 probe for | 5′-GCT CTC AAC TAC CTG CTT CC-3′ |
| Tet3 probe rev | 5′-CAT TGA GGC CAC ATC TCC G-3′ |
