# Supplementary material for: Intrinsic and Extrinsic Connections of Tet3 Dioxygenase with CXXC Zinc Finger Modules
Source: PLoS One. 2013 May 14;8(5):e62755. doi: 10.1371/journal.pone.0062755 (PMC3653909; doi:10.1371/journal.pone.0062755)
Supplement: Table S3 — Primer sequences for qPCR. (DOCX) [file pone.0062755.s012.docx]

**Table S3.** Primer sequences for Real-time PCR.

| Name | Sequence |
| --- | --- |
| Gapdh forward* | 5′-CAT GGC CTT CCG TGT TCC TA-3′ |
| Gapdh reverse* | 5′-CTT CAC CAC CTT CTT GAT GTC ATC-3′ |
| Tet1 forward* | 5′-CCA GGA AGA GGC GAC TAC GTT-3′ |
| Tet1 reverse* | 5′-TTA GTG TTG TGT GAA CCT GAT TTA TTG T-3′ |
| Tet2 forward* | 5′-ACT TCT CTG CTC ATT CCC ACA GA-3′ |
| Tet2 reverse* | 5′-TTA GCT CCG ACT TCT CGA TTG TC-3′ |
| Total Tet3 forward* | 5′-GAG CAC GCC AGA GAA GAT CAA-3′ |
| Total Tet3 reverse* | 5′-CAG GCT TTG CTG GGA CAA TC-3′ |
| Cxxc4 forward | 5′-ACC TGG CAC TTC GCT AGA GAG A-3′ |
| Cxxc4 reverse | 5′-TTG CCC TTC ATT CCC AAA TG-3′ |
| Cxxc5 forward | 5′-CAG CAG TTG TAG GAA CCG AAA GA-3′ |
| Cxxc5 reverse | 5′-TCC CGA CGG AAG CAT CAC-3′ |
| Cxxc10 forward | 5′-GTG GAG ATG GGC GGA AGA A-3′ |
| Cxxc10 reverse | 5′-GAT CTG GTG TGT GCG ACG AT-3′ |
| Tet3^CXXC^L forward | 5′-ATC GTC GCA CAC ACC AGA TC-3′ |
| Tet3^CXXC^Lreverse | 5′-TCC TTC ACG AGC ATT TAT TTC CA-3′ |
| Tet3 forward | 5′-GCG GCC GAT GCA GTA GTG-3′ |
| Tet3 reverse | 5′-ATC AAC TGG GCT GAG CTC TGA-3′ |

* Szwagierczak A, Bultmann S, Schmidt CS, Spada F, Leonhardt H. (2010) Sensitive enzymatic quantification of 5‑hydroxymethylcytosine in genomic DNA. Nucleic Acids Res., 38, e181
