# Supplementary material for: Intrinsic and Extrinsic Connections of Tet3 Dioxygenase with CXXC Zinc Finger Modules
Source: PLoS One. 2013 May 14;8(5):e62755. doi: 10.1371/journal.pone.0062755 (PMC3653909; doi:10.1371/journal.pone.0062755)
Supplement: Table S4 — Sequences of oligonucleotides used for preparation of double stranded DNA substrates. (DOCX) [file pone.0062755.s013.docx]

**Table S4.** Sequences of oligonucleotides used for preparation of double stranded DNA substrates.

M: 5-methylcytosine X: 5-hydroxymethylcytosine

| Name | Sequence |  |  |  |  |
| --- | --- | --- | --- | --- | --- |
| CGup | 5’- CTCAACAACTAACTACCATCCGGACCAGAAGAGTCATCATGG -3’ | | | |  |
| um647N | 5’- ATTO647N-CCATGATGACTCTTCTGGTCCGGATGGTAGTTAGTTGTTGAG -3’ | | | | |
| MGup | 5’- CTCAACAACTAACTACCATCMGGACCAGAAGAGTCATCATGG -3’ | | | |  |
| mC700 | 5'- ATTO700-CCATGATGACTCTTCTGGTCMGGATGGTAGTTAGTTGTTGAG -3' | | | | |
| hmCGup | 5'- CTCAACAACTAACTACCATCXGGACCAGAAGAGTCATCATGG -3' | | | |  |
| hmC550 | 5'- ATTO550-CCATGATGACTCTTCTGGTCXGGATGGTAGTTAGTTGTTGAG -3' | | | | |
| um550 | 5’- ATTO550-CCATGATGACTCTTCTGGTCCGGATGGTAGTTAGTTGTTGAG -3’ | | | | |
| um700 | 5’- ATTO700-CCATGATGACTCTTCTGGTCCGGATGGTAGTTAGTTGTTGAG -3’ | | | | |
| um590 | 5’- ATTO590-CCATGATGACTCTTCTGGTCCGGATGGTAGTTAGTTGTTGAG -3’ | | | | |
| noCGup | 5’- CTCAACAACTAACTACCATCTGGACCAGAAGAGTCATCATGG -3’ | | | | |
| noCG647N | 5’- ATTO647N-CCATGATGACTCTTCTGGTCTGGATGGTAGTTAGTTGTTGAG -3’ | | | | |
