# Supplementary material for: Intrinsic and Extrinsic Connections of Tet3 Dioxygenase with CXXC Zinc Finger Modules
Source: PLoS One. 2013 May 14;8(5):e62755. doi: 10.1371/journal.pone.0062755 (PMC3653909; doi:10.1371/journal.pone.0062755)
Supplement: Table S5 — CG, mCG and hmCG containing DNA substrates used for in vitro binding assay (related to Fig. 5 ). (DOCX) [file pone.0062755.s014.docx]

**Table S5.** CG, mCG and hmCG containing DNA substrates used for *in vitro* binding assay (referes to Fig. 5).

|  | Name | CpG site | Label | Oligo I | Oligo II |
| --- | --- | --- | --- | --- | --- |
| sample set | 647N-CG | unmethylated | 647N | CGup | um647N |
|  | 700-mC | fully methylated | 700 | MGup | mC701 |
|  | 550-hmC | fully hydroxymethylated | 550 | hmCGup | hmC550 |
| control set | 647N-CG | unmethylated | 647N | CGup | um647N |
|  | 550-CG |  | 550 |  | um550 |
|  | 700-CG |  | 700 |  | um700 |
