# Supplementary material for: Intrinsic and Extrinsic Connections of Tet3 Dioxygenase with CXXC Zinc Finger Modules
Source: PLoS One. 2013 May 14;8(5):e62755. doi: 10.1371/journal.pone.0062755 (PMC3653909; doi:10.1371/journal.pone.0062755)
Supplement: Table S6 — CG and noCG containing DNA substrates used for in vitro binding assay (related to Fig. S7). (DOCX) [file pone.0062755.s015.docx]

**Table S6.** CG and noCG containing DNA substrates used for *in vitro* binding assay (referes to Fig. S7).

|  | Name | CpG site | Label | Oligo I | Oligo II |
| --- | --- | --- | --- | --- | --- |
| sample set | 590-CG | ummethylated | 590 | CGup | um590 |
|  | 647N-noCG | no CpG site | 647N | noCGup | noCG647N |
| control set | 590-CG | unmethylated | 590 | CGup | um590 |
|  | 647N-CG |  | 647N |  | um647N |
