# Supplementary material for: Intrinsic and Extrinsic Connections of Tet3 Dioxygenase with CXXC Zinc Finger Modules
Source: PLoS One. 2013 May 14;8(5):e62755. doi: 10.1371/journal.pone.0062755 (PMC3653909; doi:10.1371/journal.pone.0062755)
Supplement: Combined Supporting Information File S1 — (PDF) [file pone.0062755.s016.pdf]

Intrinsic and extrinsic connections of Tet3 dioxygenase with CXXC zinc finger modules

Nan Liu, Mengxi Wang, Wen Deng, Christine S. Schmidt, Weihua Qin, Heinrich Leonhardt and Fabio Spada

Department of Biology II, Ludwig Maximilians University Munich, Planegg-Martinsried, Germany.

SUPPORTING INFORMATION FILE

Figures S1-9.

Tabels S1-6.

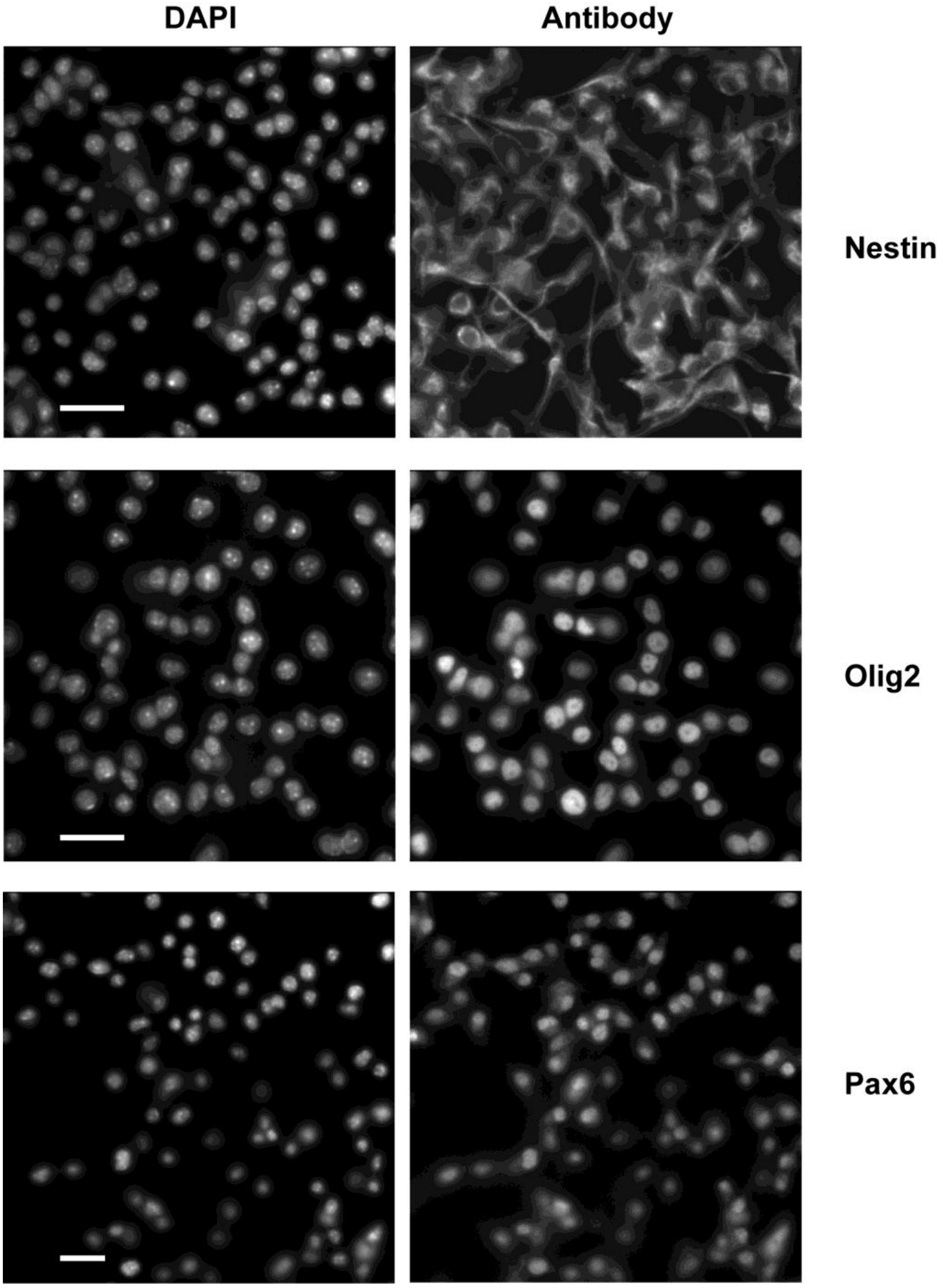

Figure S1. Expression of NSCs markers in ENC1 cells. Epifluorescence images of immunofluorescent stainings with antibodies to the indicated markers. Antibody sources: Nestin, mouse monoclonal antibody Rat-401 (Developmental Studies Hybridoma Bank, University of Iowa); Pax6, rabbit polyclonal antibody (PRB-278P, Covance). Olig2, rabbit polyclonal antibody (AB9610, Millipore). Scale bars: 10 µm.

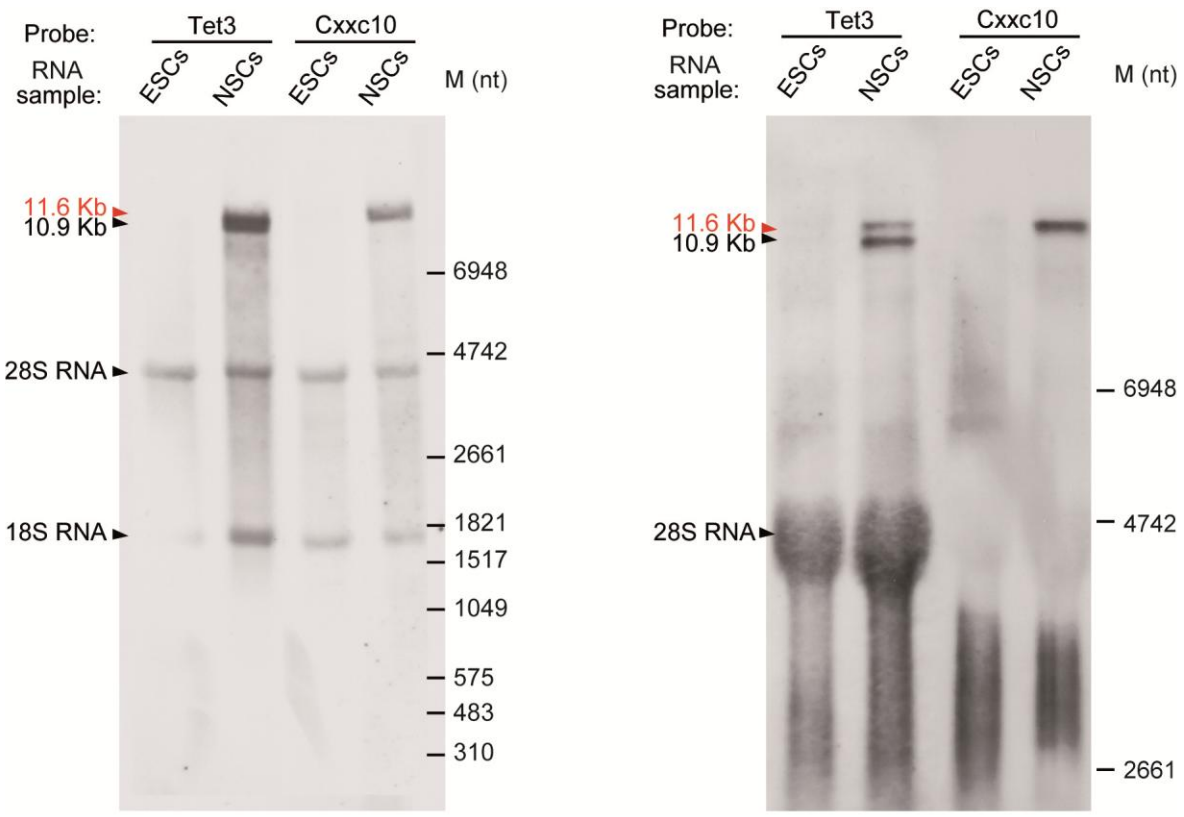

Figure S2. Northern blot analysis of Tet3 and Tet3^{CXXC}L transcripts in NSCs and ESCs (related to Fig. 2). On the right the same blot as in Fig. 2D is shown uncropped. In this blot total RNA was loaded [without poly(A)$^{+}$ enrichment], resulting in stronger crosshybridization with 28S and 18S ribosomal RNAs.

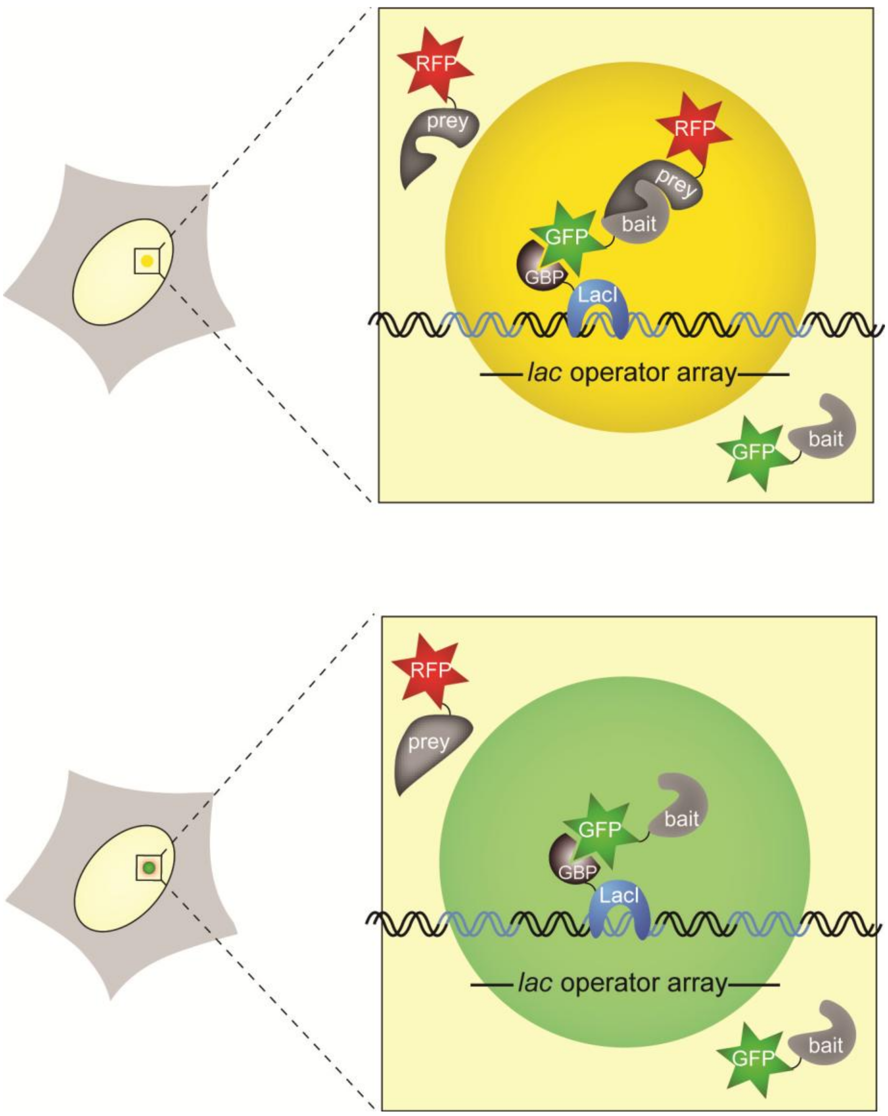

Figure S3. Schematic representation of the mammalian F3H assay (related to Fig. 4).

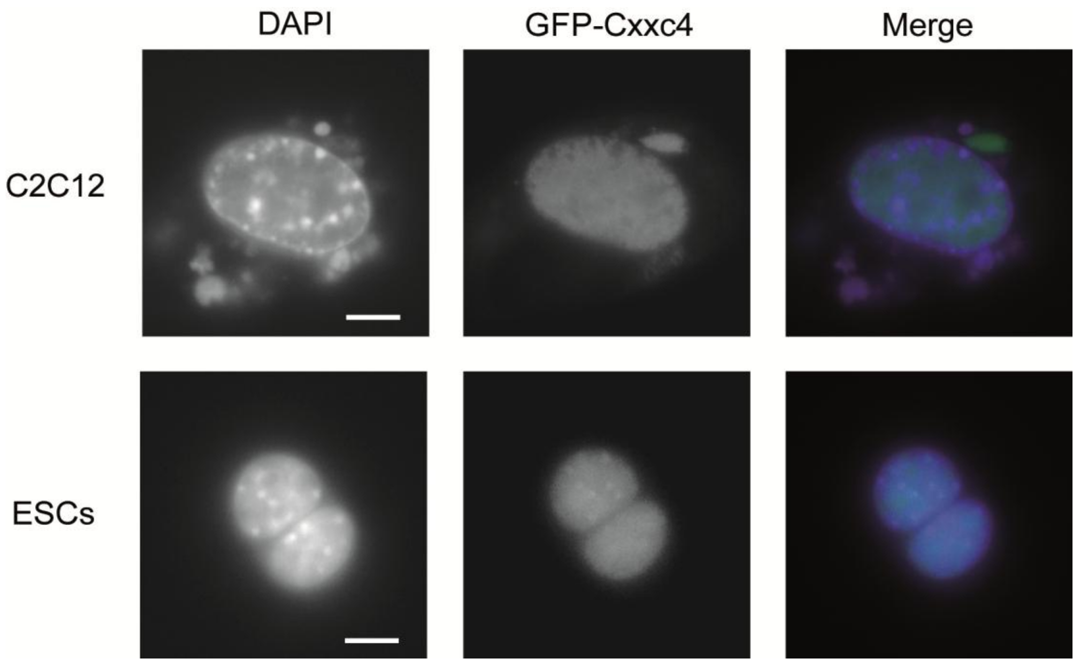

Figure S4. Nuclear localization of GFP-Cxxc4 in C2C12 myoblasts and CGR8 ESCs (related to Fig. 4). Epifluorescence images of transiently transfected cells. Scale bars: 5 µm.

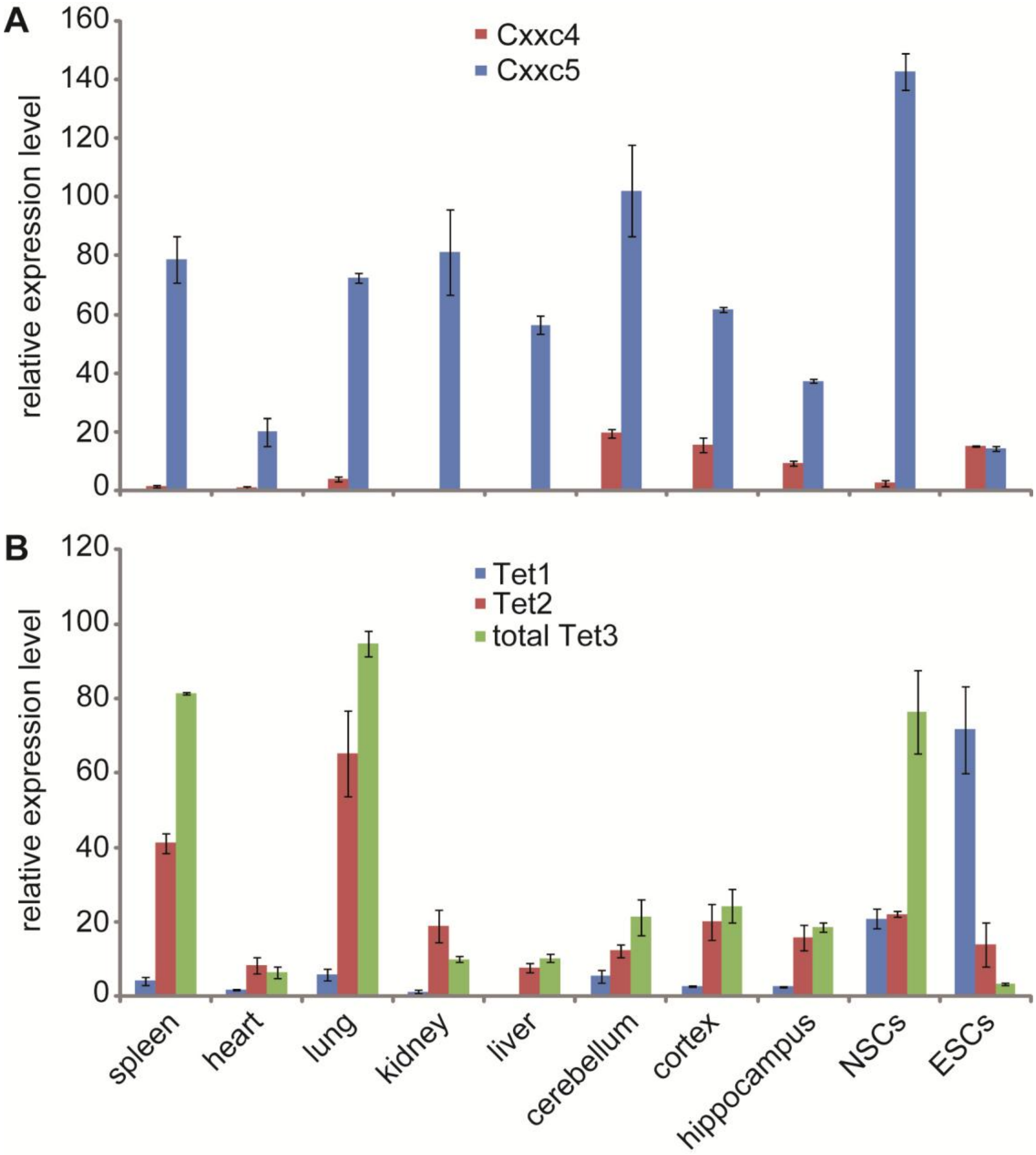

Figure S5. Transcript levels of Cxxc4, Cxx5 (A) and Tet1-3 (B) in adult mouse tissues ESCs and NSCs (related to Fig. 3). In (A) the same plot as in Fig. 3B is reported for ease of comparison between transcript levels of Cxxc4/5 (A) and Tet1-3 (B). In (B) cumulative levels of all Tet3 transcripts were determined using a primer set spanning common sequences downstream exon 3 of the annotated *Tet3* gene. Shown are mean values and SEM. Sample sources and replicates are as for Fig. 3.

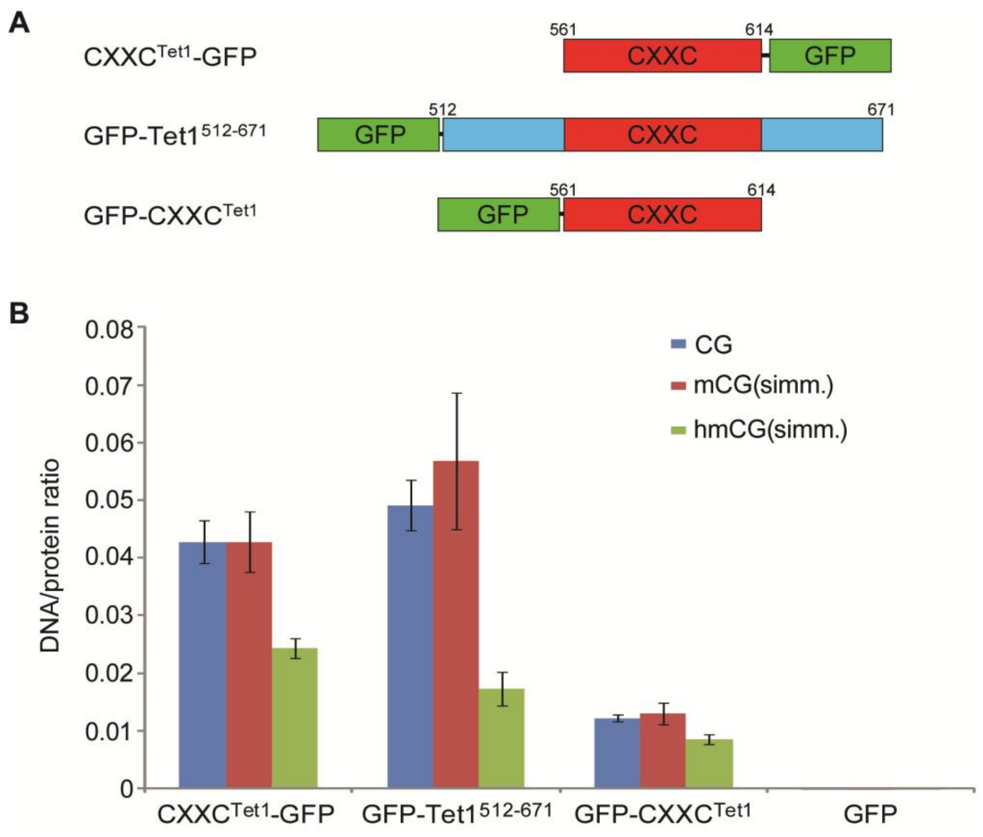

Figure S6. *In vitro* DNA binding properties of GFP-Tet1$^{512\text{-}671}$, GFP-CXXC^{Tet1} and CXXC^{Tet1}-GFP. (A) Schematic representation of assayed Tet1 constructs. Start and end positions relative to full length Tet1 protein are reported. (B) DNA binding assay as in Fig. 5. Shown are mean values and SEM from 4 independent experiments.

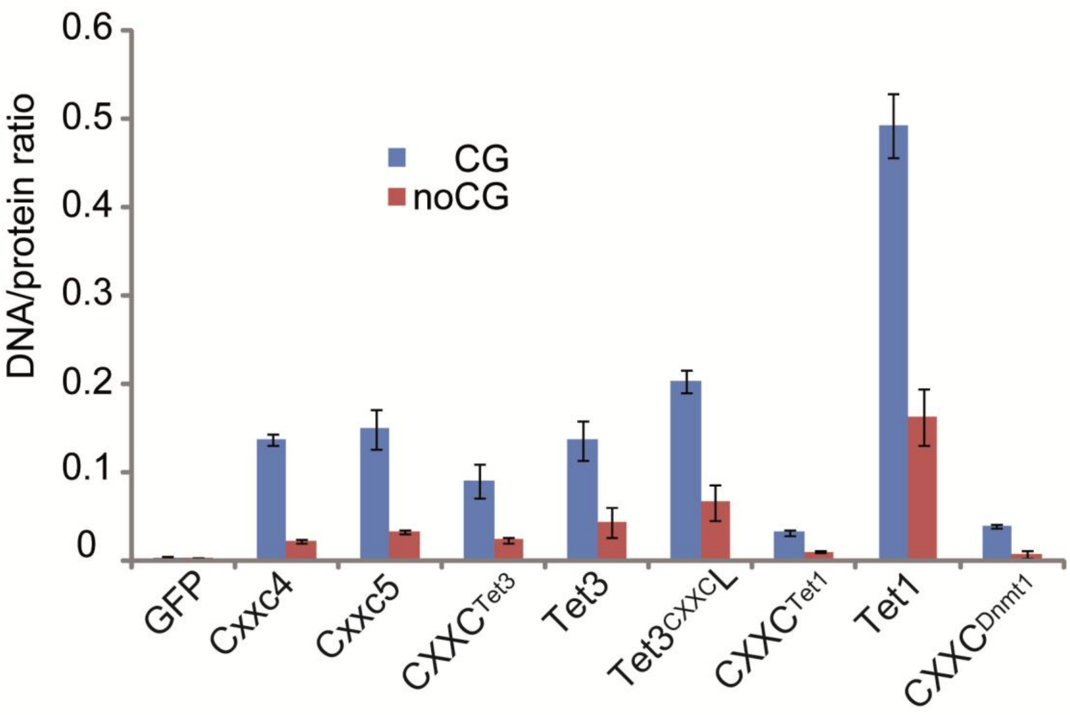

Figure S7. *In vitro* binding of various full length CXXC domain-containing proteins and isolated CXXC domains to DNA substrates containing one or no CpG site (noCG; TpG instead of CpG), but otherwise identical sequence (related to Fig. 5). All constructs are GFP fusions. Shown are mean values of bound substrate/protein ratios and SEM from n independent replicate experiments: GFP and CXXC^{Tet3}-GFP, n=5; GFP-Tet1, Cxxc4-GFP, Cxxc5-GFP and GFP-CXXC^{Dnmt1}, n=4; GFP-Tet3, GFP-Tet3^{CXXC}L and CXXC^{Tet1}-GFP, n=3.

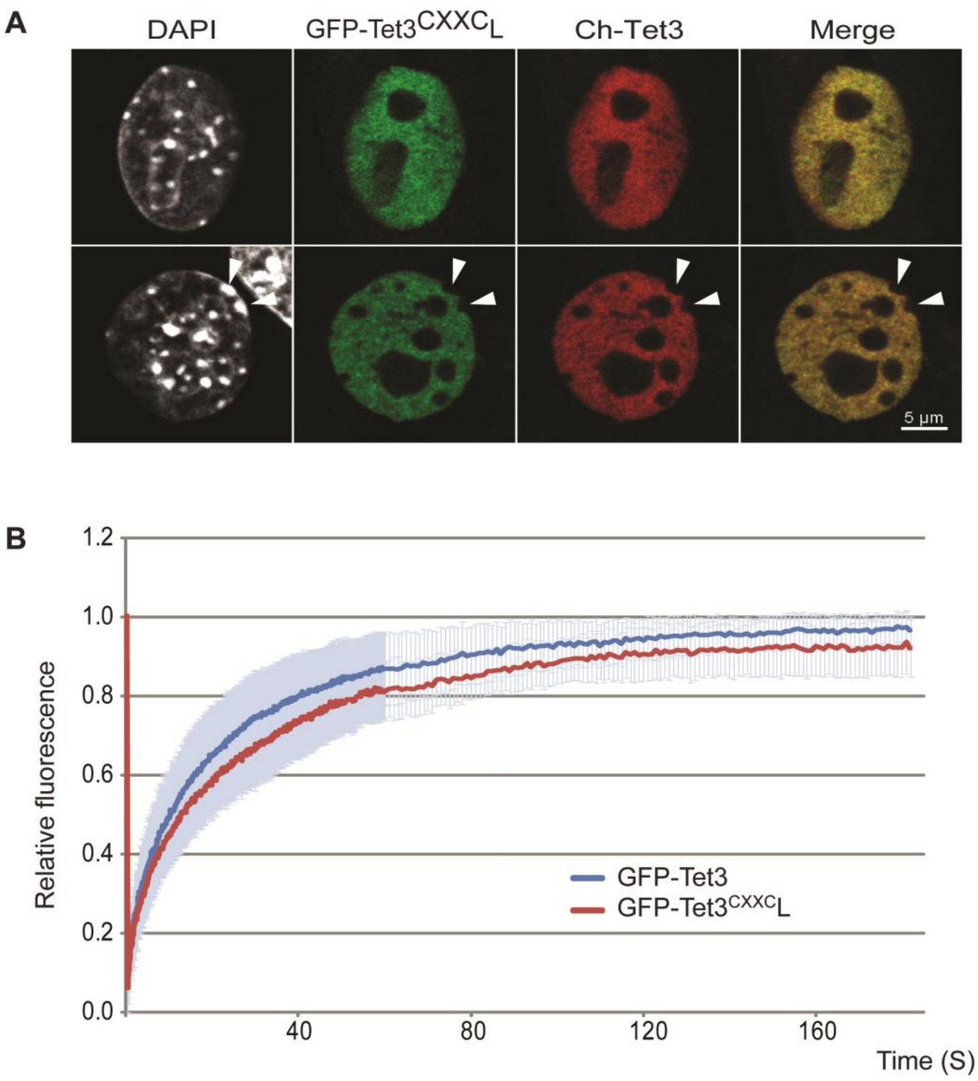

Figure S8. Localization (A) and mobility (B) of Tet3 and Tet3^{CXXC}L isoforms in C2C12 nuclei. (A) Optical sections of fixed C2C12 cotransfected with GFP-Tet3^{CXXC}L and Ch-Tet3 constructs as indicated. Arrowheads indicate the position of large chromocenters from which GFP-Tet3^{CXXC}L and Ch-Tet3 signals are excluded. (B) FRAP curves of GFP-Tet3 and GFP-Tet3^{CXXC}L in transiently transfected C2C12 myoblasts. Images were taken every 150 ms in the first 60 s, and then at intervals of 1 s for the next 120 s. Shown are mean values and SEM from 12 (GFP-Tet3) and 10 cells (GFP-Tet3^{CXXC}L). Live cell imaging and FRAP analysis was performed as described (Schermelleh et al., 2007, Nucl Acids Res 35: 4301) with the following minor modifications. The images were Gauss-filtered (2 pixel radius) and data sets showing lateral movement were corrected by image registration using the StackReg plug-in of ImageJ, starting with a time frame where approximately half recovery was reached.

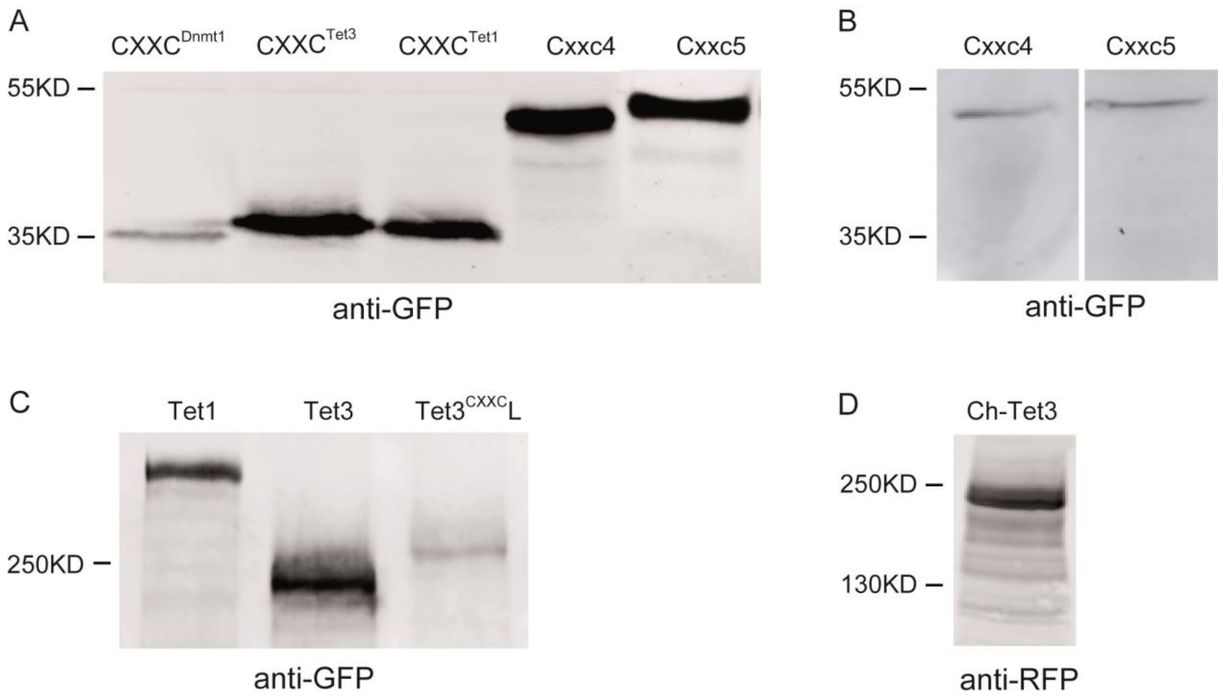

Figure S9. Western blot analysis of fluorescent fusion proteins. (A) GFP-CXXC^{Dnmt1}, CXXC^{Tet3}-GFP, CXXC^{Tet1}-GFP, Cxxc4-GFP, Cxxc5-GFP. (B) GFP-Cxx4 and GFP-Cxxc5. (C) GFP-Tet1, GFP-Tet3 and GFP-Tet3^{CXXC}L. (D) Cherry-Tet3. Blots were probed with an anti-GFP antibody (A-C) or with an anti-RFP antibody recognizing an epitope present in both RFP and Cherry (D). In all cases the major reacting band migrated as a peptide with the expected mass of the specific, full length fluorescence fusion and in no case peptides with mass corresponding to the fluorescent protein moiety (GFP or Cherry) were detected.

Table S1. Primers for amplification and insertion of coding sequences in expression constructs.

Construct	Primer
$Tet1^{512\text{-}671}$	5’-AAG CGA TCG CTT AGA TCT TAC CCA GGG-3’
	5’-TTG CGG CCG CCA AAT CCA ACC TTT GC-3’
$CXXC^{Tet1}$	5’-GGC GAT CGC ATG TCT ACG CCG CCA ATG-3’
	5’-CGC GGC CGC CTG GCT TCT TTT TGA GCA-3’
Cxxc4	5′-ATG CAC CAC CGG AAC GAC TCC CAG CG-3’
	5’-TTA AAA GAA CCA TCG GAA CGC TTC AGC-3’
Cxxc5	5′-AAG CGA TCG CAT GTC GAG CCT CGG CGG TGG-3′
	5′-GCG CGG CCG CTC ACT GAA ACC ACC GGA AGG-3′
$CXXC^{Tet3}$	5′-ATG CGA TCG CAT GCT GCG AGG GGG TGG AGA T-3′
	5′-ATG CGG CCG CCC GCT TTT TTC TTC AGC ACC TC-3′
$Tet3^{CXXC}L$	5′-GGG CGA TCG CAT GAG CCA GTT TCA GGT GCC CTT GG-3′
	5′-GCG GCC GCC TAG ATC CAG CGG CTG TAG GGG CC-3′

Table S2. Primer sequences for 5′ RACE, conventional RT-PCR (primers a-d indicated in Fig. 2A,B) and generation of probes for northern blotting.

Name	Sequence
GSP1	5′ -AGG TCC ATC AAC TGG GCT-3′
$(dT)_{17}$-adaptor	5′-GAC TCG AGT CGA CAT CGA $(T)_{17}$-3′
adaptor primer	5′-GAC TCG AGT CGA CAT CG-3′
GSP2	5′-AGC ACC TCA CAC TTG CG-3′
GSP3	5′-GCA GCT GGT ACA AGA CC-3′.
Primer a	5′- GCG ATC GCA TGA GCC AGT TTC AGG -3′
Primer c	5′- AAG CGG CCG CCA GTC GGG CTT CTG GTC TAC -3′
Primer b	5′- ATG GCT GGG AGT GAG AC -3′
Primer d	5′- ATC GCA GGT GCA GTT GGG TG -3′
CXXC10 probe for	5′-CAC ACC CAT TGG CTC ACC T-3′
CXXC10 probe rev	5′-GGG TCT CAC TCC CAG CCA-3′
Tet3 probe for	5′-GCT CTC AAC TAC CTG CTT CC-3′
Tet3 probe rev	5′-CAT TGA GGC CAC ATC TCC G-3′

Table S3. Primer sequences for Real-time PCR.

Name	Sequence
Gapdh forward*	5′-CAT GGC CTT CCG TGT TCC TA-3′
Gapdh reverse*	5′-CTT CAC CAC CTT CTT GAT GTC ATC-3′
Tet1 forward*	5′-CCA GGA AGA GGC GAC TAC GTT-3′
Tet1 reverse*	5′-TTA GTG TTG TGT GAA CCT GAT TTA TTG T-3′
Tet2 forward*	5′-ACT TCT CTG CTC ATT CCC ACA GA-3′
Tet2 reverse*	5′-TTA GCT CCG ACT TCT CGA TTG TC-3′
Total Tet3 forward*	5′-GAG CAC GCC AGA GAA GAT CAA-3′
Total Tet3 reverse*	5′-CAG GCT TTG CTG GGA CAA TC-3′
Cxxc4 forward	5′-ACC TGG CAC TTC GCT AGA GAG A-3′
Cxxc4 reverse	5′-TTG CCC TTC ATT CCC AAA TG-3′
Cxxc5 forward	5′-CAG CAG TTG TAG GAA CCG AAA GA-3′
Cxxc5 reverse	5′-TCC CGA CGG AAG CAT CAC-3′
Cxxc10 forward	5′-GTG GAG ATG GGC GGA AGA A-3′
Cxxc10 reverse	5′-GAT CTG GTG TGT GCG ACG AT-3′
Tet3^{CXXC}L forward	5′-ATC GTC GCA CAC ACC AGA TC-3′
Tet3^{CXXC}Lreverse	5′-TCC TTC ACG AGC ATT TAT TTC CA-3′
Tet3 forward	5′-GCG GCC GAT GCA GTA GTG-3′
Tet3 reverse	5′-ATC AAC TGG GCT GAG CTC TGA-3′

* Szwagierczak A, Bultmann S, Schmidt CS, Spada F, Leonhardt H. (2010) Sensitive enzymatic quantification of 5-hydroxymethylcytosine in genomic DNA. Nucleic Acids Res., 38, e181

Table S4. Sequences of oligonucleotides used for preparation of double stranded DNA substrates.

M: 5-methylcytosine X: 5-hydroxymethylcytosine

Name	Sequence
CGup	5'- CTCAACAACTAACTACCATCCGGACCAGAAGAGTCATCATGG -3'
um647N	5'- ATTO647N-CCATGATGACTCTTCTGGTCCGGATGGTAGTTAGTTGTTGAG -3'
MGup	5'- CTCAACAACTAACTACCATCMGGACCAGAAGAGTCATCATGG -3'
mC700	5'- ATTO700-CCATGATGACTCTTCTGGTCMGGATGGTAGTTAGTTGTTGAG -3'
hmCGup	5'- CTCAACAACTAACTACCATCXGGACCAGAAGAGTCATCATGG -3'
hmC550	5'- ATTO550-CCATGATGACTCTTCTGGTCXGGATGGTAGTTAGTTGTTGAG -3'
um550	5'- ATTO550-CCATGATGACTCTTCTGGTCCGGATGGTAGTTAGTTGTTGAG -3'
um700	5'- ATTO700-CCATGATGACTCTTCTGGTCCGGATGGTAGTTAGTTGTTGAG -3'
um590	5'- ATTO590-CCATGATGACTCTTCTGGTCCGGATGGTAGTTAGTTGTTGAG -3'
noCGup	5'- CTCAACAACTAACTACCATCTGGACCAGAAGAGTCATCATGG -3'
noCG647N	5'- ATTO647N-CCATGATGACTCTTCTGGTCTGGATGGTAGTTAGTTGTTGAG -3'

Table S5. CG, mCG and hmCG containing DNA substrates used for *in vitro* binding assay (referes to Fig. 5).

	Name	CpG site	Label	Oligo I	Oligo II
sample set	647N-CG	unmethylated	647N	CGup	um647N
	700-mC	fully methylated	700	MGup	mC701
	550-hmC	fully hydroxymethylated	550	hmCGup	hmC550
control set	647N-CG	unmethylated	647N	CGup	um647N
	550-CG		550		um550
	700-CG		700		um700

Table S6. CG and noCG containing DNA substrates used for *in vitro* binding assay (referes to Fig. S7).

	Name	CpG site	Label	Oligo I	Oligo II
sample set	590-CG	ummethylated	590	CGup	um590
	647N-noCG	no CpG site	647N	noCGup	noCG647N
control set	590-CG	unmethylated	590	CGup	um590
	647N-CG		647N		um647N
